# Impact of advance care planning on the care of patients with heart failure: study protocol for a randomized controlled trial

**DOI:** 10.1186/s13063-016-1414-1

**Published:** 2016-06-10

**Authors:** Chetna Malhotra, David Kheng Leng Sim, Fazlur Jaufeerally, Nivedita Nadkarni Vikas, Genevieve Wong Cheng Sim, Boon Cheng Tan, Clarice Shu Hwa Ng, Pei Leng Tho, Jingfen Lim, Claire Ya-Ting Chuang, Florence Hui Mei Fong, Joy Liu, Eric A. Finkelstein

**Affiliations:** Lien Centre for Palliative Care, Duke-NUS Medical School, 8 College Road, Singapore, 169857 Singapore; National Heart Centre Singapore, 5 Hospital Drive, Singapore, 169609 Singapore; Singapore General Hospital, Outram Road, Singapore, 169608 Singapore; Duke-NUS Medical School, 8 College Road, Singapore, 169857 Singapore

**Keywords:** Advance care planning, End-of-life, Preferences, Heart failure

## Abstract

**Background:**

Despite the promise and popularity of advance care planning, there is insufficient evidence that advance care planning helps patients to meet their end-of-life care preferences, especially in Asian settings. Thus, the proposed study aims to assess whether patients with advanced heart failure who are receiving advance care planning have a greater likelihood of receiving end-of-life care consistent with their preferences compared to patients receiving usual care. Secondary objectives are to compare differences in health care expenditures, quality of life, anxiety and depression, understanding of own illness, participation in decision-making and concordance with their caregiver’s preferences for end-of-life care, between patients with advanced heart failure receiving advance care planning and usual care.

**Methods/design:**

This is a two-arm randomized controlled trial of advance care planning versus usual care (control) conducted at two institutions in Singapore. Two hundred and eighty-two patients with advanced heart failure (*n* = 94 in the advance care planning arm; *n* = 188 in the control arm receiving usual care) will be recruited from these centers and followed for 1 year or until they die, whichever is earlier. Additionally, the study will include up to one caregiver per patient enrolled.

**Discussion:**

If advance care planning is proven to be effective, the results will help to promote its uptake among health care providers and patients both within Singapore and in other countries.

**Trial registration:**

NCT02299180. Registered on 18 November 2014.

## Background

Worldwide there is an increasing awareness of inadequacy in end-of-life (EOL) care provided to patients with non-cancer-related terminal illnesses, such as advanced congestive heart failure (CHF) [1]. Data from several countries shows that despite therapeutic advances and high health care expenditures [2], patients with advanced CHF suffer enormously from the physical and psychological effects of their illness [3–5]. Unlike many patients with advanced cancer, patients with CHF typically experience a gradual but non-linear decline in physical function over many months or years, with occasional periods of exacerbation [6]. The non-linear deterioration in health leads to difficulties in prognostication and often delayed referral to specialist palliative care services. Moreover, there is evidence that patients’ understanding of their illness is often poor and communication with their physician inadequate [7, 8]. These concerns suggest that patients may be receiving suboptimal EOL care that is not consistent with their preferences and receiving EOL care that may involve disproportionately large health care expenditures.

There is increasing evidence that the presence of advance directives alone does not help in meeting patient preferences or in reducing aggressive treatments and cost of care [9–13]. In contrast, advance care planning (ACP), one of the most widely discussed and studied of all EOL conversations, is believed to enable patients to meet their EOL preferences. This is because ACP is an ongoing process by which the patient, in consultation with health care providers and/or family members/caregivers, makes decisions about their future health care should they become incapable of participating in medical treatment decisions. Further, it shifts the focus on EOL decision-making away from completion of documents and towards facilitating discussion about values and preferences regarding treatment and care, and involves family members in the discussion [9].

Despite the promise and popularity, there has been little research evaluating the effectiveness of ACP in meeting patient preferences, especially in Asian settings. One randomized controlled trial (RCT) of the “Respecting Choices” model for ACP, among elderly hospitalized patients in Australia, has reported positive outcomes in enabling people to receive EOL treatment as per their wishes [14]. Another controlled trial, conducted in a nursing home in the US also found higher compliance with patients’ wishes among those receiving ACP [15]. An RCT by Kirchhoff et al. [16] among individuals with CHF or end-stage renal disease failed to show any significant difference in the proportion of patients who met their preferences regarding cardiopulmonary resuscitation (CPR) between the ACP and control arms. Thus, there has been mixed evidence regarding the effectiveness of ACP, with none from an Asian setting.

This study aims to fill this gap. It is a two-arm RCT of patients with advanced CHF to assess the impact of ACP on health and cost outcomes. The study will be conducted in Singapore, a rapidly aging country in Southeast Asia. We will survey the patients in both arms every 4 months for a period of 1 year or until they die, whichever is earlier. The primary objective of this trial is to assess whether patients receiving ACP have a greater likelihood of receiving EOL care consistent with their preferences compared to patients receiving usual care. Secondary objectives are to compare heath care expenditures, quality of life, anxiety and depression, understanding of own illness, participation in decision-making and concordance with caregiver preferences for EOL care between patients receiving ACP and usual care. We hypothesize that patients receiving ACP will be more likely to receive EOL care consistent with their stated preferences, and have lower health care expenditures, better quality of life, lower anxiety and depression, better understanding of their own illness, greater participation in decision-making, and greater congruence with caregiver preferences, as compared to patients receiving usual care.

## Methods/design

### Study design

This is a two-arm RCT of ACP versus usual care conducted at two institutions in Singapore –the National Heart Centre Singapore and Singapore General Hospital (Department of Internal Medicine). The two hospitals were chosen because they are the largest centers/hospitals that currently have trained ACP facilitators to deliver ACP to patients with advanced CHF. All ACP facilitators in Singapore are trained based on similar principles and with similar intensity and methodology. Therefore, the study results are likely to be generalizable to patients with advanced CHF who are receiving ACP at any hospital in Singapore.

Two hundred and eighty-two in-patients with advanced CHF (*n* = 94 in the ACP arm; *n* = 188 in the control arm receiving usual care) will be recruited from these centers and followed for 1 year or until they die, whichever is earlier. Additionally, the study will include up to one caregiver per patient enrolled.

Randomization will follow a 1:2 (ACP: control arm) ratio. The unequal number of patients in the ACP and control arms is because it was anticipated that over the course of the study duration, due to increasing popularity of ACP in Singapore, many patients in the control arm may decide to take up ACP, thus contaminating the control arm. If this happens, the larger number of participants in the control group is expected to increase the statistical power for a “per-protocol” analysis [17].

### Study population

The study will recruit patients with advanced CHF (New York Heart Association classes III and IV) while they are hospitalized, who are 21 years and older and able to give informed consent. Those with psychiatric or cognitive disorders, a previously documented ACP or those who have undergone ACP facilitation will be excluded. The study protocol has been approved by the Institutional Review Board at Singhealth.

### Intervention

The patient and their family members will be referred to an ACP facilitator. The ACP facilitator will be certified in providing ACP. Other than assisting individuals in determining their preferences for future medical care, the ACP facilitator will provide emotional support to patients/families with making EOL decisions. Family members will be encouraged to be present during the ACP discussion so that the whole family unit will be able to explore goals, values and beliefs towards the patient’s medical care. With family members’ participation, there is a greater likelihood that they come to understand and support the decisions made by the patient during ACP. Patients will be encouraged to appoint a substitute health care decision-maker who will make the decisions on their behalf when they are no longer able to do so.

The ACP document is dated and specifies whether the patient prefers CPR or not, and whether the patient wants comfort measures only (medications, oxygen and other measures may be used for comfort at the place where the patient lives), limited additional intervention (may include limited trial of treatment, oral/intravenous medications, non-invasive ventilation support and transfer to hospital, if needed) or full treatment (may consider intubation, mechanical ventilation, cardioversion and transfer to intensive care, if needed) as well as any other additional care preferences. Preferred place of medical treatment in the event of deterioration as well as preferred place of death is also noted in the ACP document. The names of substitute decision-makers are specified. A copy of the completed ACP document will be provided to the patient, substitute health care decision-makers and the health care team. The ACP document will be filed onto the front page of the patient’s inpatient case notes and a copy will be scanned into the patient’s electronic medical records in the National IT System, which is shared by all hospitals in Singapore, for easy retrieval and viewing in case the patient is admitted to any hospital in Singapore. This is to ensure that immediate retrieval of the ACP document is possible when treatment decisions must be made quickly.

Patient preferences among those in the intervention arm will be reviewed periodically upon readmission or during the outpatient clinic by the ACP facilitator. If the patient expresses different preferences towards future medical care, then the ACP facilitator will conduct a session as soon as possible to explore the updated goals for the patient’s current medical preferences and the existing ACP document will be voided. If the patient has a change of mind towards future medical care in between visits, then the patient will be encouraged to contact the ACP facilitator in charge via phone of their change in mind. The ACP facilitator will then arrange for a session as soon as possible to explore the patient’s wishes and update the ACP document.

### Control arm

The control arm patients will not take part in ACP discussions and documentation, but will continue to receive usual care.

### Outcome measures

#### Primary outcome

The primary outcome to be assessed in the study is the proportion of patients receiving EOL care consistent with their stated preferences. Patients’ stated preferences for CPR, life-prolonging treatments (comfort measures only/limited intervention/full treatment), place of care and place of death will be assessed from their last survey or their ACP document. The actual treatment received by the patient will be assessed from their medical records after the patient’s death. If these records are unavailable, this information will be assessed in post-death interviews with the patient’s family members/surrogate decision-maker. Actual place of death will be ascertained from death certificates and actual place of care from post-death interviews with the patient’s family members/surrogate decision-maker. The analysis will only be conducted for the sub-group of patients who die during the study period.

Secondary outcomes:

1. Heath care expenditures during study duration: inpatient costs will be assessed through linkages with hospital billing records
2. Patients’ quality of life: patients’ quality of life will be measured through Kansas City Cardiomyopathy Questionnaire (KCCQ). The KCCQ is a 23-item questionnaire that quantifies physical limitations, symptoms, self-efficacy, social interference and quality of life for patients with CHF [18]. This scale will be administered both during baseline and follow-up survey
3. Patients’ anxiety and depression: we will assess patients’ anxiety and depression through the Hospital Anxiety and Depression Scale (HADS) [19, 20]. HADS is an extensively used, valid and reliable scale to measure anxiety and depression among patients in non-psychiatric hospital clinics. It has two subscales – an anxiety subscale (HADS-A) and a depression (HADS-D) subscale, with a total score ranging from 0 to 21 for each. Internal consistency (Cronbach’s alpha) for HADS-A varies from .68 to .93 and for HADS-D from .67 to .90. An optimal balance of sensitivity and specificity is achieved by a score of 8 or above on both HADS-A and HADS-D. The sensitivity and specificity for both HADS-A and HADS-D is 0.80 [19]. These scales will be administered during the baseline interview and during follow-up surveys with the patient
4. Patients’ understanding of own illness: understanding of illness will be assessed by asking patients whether they thought that their current treatments would help them to live longer (yes/no/unsure), cure their heart condition (yes/no/unsure) and relieve their symptoms (yes/no/unsure)
5. Patients’ participation in decision-making: patients’ participation in decision-making will be measured by the Decisional Conflict Scale [21] administered at baseline and in follow-up surveys. The Decisional Conflict Scale measures patient’s state of uncertainty about the course of action to take. This state is likely to occur when making choices involving risk or uncertainty of outcomes and the need to make tradeoffs in selecting a course of action (e.g., when making decisions regarding EOL treatment and care). The scale’s internal consistency ranges from 0.78 to 0.92 and the test-retest reliability coefficient is .81 [21]. It has also been used and validated among cancer patients [22]
6. Patient and caregiver congruence in EOL preferences: for patients whose caregivers also consent to participate in the survey, we will assess their preferences for patient’s EOL care – CPR, life-prolonging treatments and place of death

### Recruitment and data collection

A list of patients with heart failure who are admitted at each center will be sent to the research team daily by heart failure nurses/research coordinators from these centers. The research team will then screen the list for eligibility criteria and approach the patient and family members for participation in the study; those agreeable to participate will be administered a written informed consent form and administered the baseline survey.

Eligible patients who consent to participate in the RCT and answer the survey questionnaires will be randomized to the ACP and control arms. Participants who are randomized into the intervention (ACP) arm will meet with ACP facilitators within 48 hours of consent for intervention delivery. Patients randomized to the control arm will not take part in ACP discussions and documentation, but will continue to receive usual care.

Randomization will be done using block randomization by generating a random allocation sequence of participants to the intervention and control arms through a computerized random number generator. This will be done by a statistician at the institution. The allocation will be concealed in sequentially numbered, opaque and sealed envelopes by the data manager and given to the research coordinator conducting patient recruitment at the two centers. Envelopes will be opened sequentially by the research coordinator only after the participant unique ID is written on the envelope. Randomization sequence will not be known to the research coordinator, health care providers (physicians, ACP facilitators, heart failure nurses) and other researchers involved in the study.

**Fig. 1:**
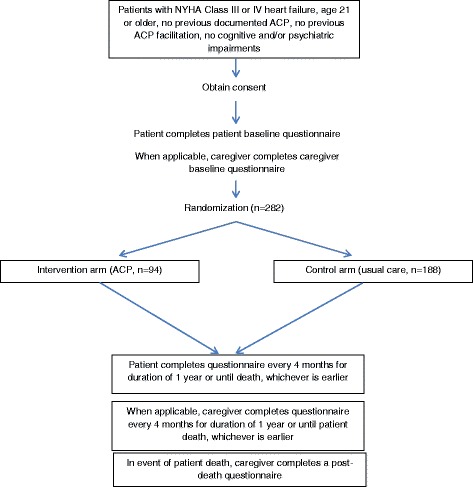
Study procedures

Patients will be followed by the research team for 1 year or until death, whichever is earlier. Further follow-up may be needed if the targeted number of deaths in the intervention and control arms is not reached within the 1-year follow-up period. Patients will be interviewed first at baseline and then every 4 months. At the end of the follow-up period, their medical and billing records will be reviewed to assess the treatment they received and related costs. All questionnaires for both patients and caregivers will be administered in their preferred language from a selection of four: English, Mandarin, Malay, and Tamil. If the patient dies during the study, consenting caregivers will be asked to complete a post-death questionnaire (Fig. 1).

Patient particulars and details of their next follow-up will be stored on a secure database to be built before the commencement of the study. This database will send text reminders to patients and their interviewers before every follow-up visit. Unique log-in for staff will be required for access.

### Sample size

Similar to a previous RCT, [14] we assumed that the proportion of patients receiving EOL care consistent with their stated preferences in the control arm will be 15 % compared to 65 % in the intervention arm. To achieve 90 % power to detect a difference in outcome between arms with a certainty of 95 %, we estimated that we will need 14 deaths in the intervention arm and 28 deaths in the control group. Assuming a mortality of about 15 % in 1 year, we will require at least 94 patients in the intervention arm and 188 patients in the control group. If the total number of deaths in the intervention and control arms is not reached during the planned 1-year follow-up period, further follow-ups will be needed.

### Proposed statistical analysis

All analysis will be done as per the intention-to-treat principle. In addition, a “per-protocol” analysis will also be conducted. For the analysis of primary outcome, we will use the kappa statistic to assess congruence between preferred and actual place of death and for each of the life-sustaining treatments. The chi-square test or Fisher’s exact test as appropriate will be used for testing the association between receiving care consistent with patients stated preference and the type of intervention. The analysis will be conducted in the sub-group of patients who die during the study duration.

For comparing health care expenditures between the two arms, we will use ordinary least squares regression to model health care expenditures with the type of intervention as the primary predictor. The model will adjust for any differences in baseline characteristics between the two arms and primary diagnosis. If health care expenditure data are not normally distributed, appropriate transformations or two-part models will be used.

For comparing quality of life, anxiety and depression, understanding of own illness and participation in decision-making, we will use separate mixed-effects models to analyze the changes over time and determine if change over time differs between the intervention and control arms. We will adjust for differences in baseline characteristics between the intervention and control arms in the model. Missing observations will be accounted for under the missing-at-random assumption of the mixed-effects model. This is a reasonable and common assumption. If the data are not normally distributed, appropriate transformations will be attempted before resorting to the use of nonparametric statistical analysis methods. Finally, the kappa statistic will be used to test the congruence between the patient’s preference and the caregiver’s preference in the analysis of the secondary outcome.

#### Qualitative in-depth interviews

We will conduct qualitative in-depth interviews with the health care staff involved in providing ACP and with family members/nominated surrogates of deceased patients. In these in-depth interviews, we will examine their views and experiences regarding ACP and why they believe ACP is successful or not in meeting patient preferences for EOL care. This will enable us to identify the strengths and limitations of ACP in Singapore and in tailoring the intervention to address limitations and replicate it in other health care institutions in Singapore and other countries.

## Discussion

This study assesses the impact of ACP in meeting preferences for EOL care among patients with advanced CHF. Until now, this outcome has been evaluated only in one RCT by Detering et al. [14]. However, that RCT was conducted among elderly hospitalized patients with varied diagnoses and not specifically among patients with advanced CHF [14]. Further, we do not know whether the beneficial effect of ACP is also applicable to Asian settings where family caregivers and physicians mostly make decisions for the patient. In addition, the present study will also assess whether ACP lowers health care expenditures, improves quality of life, decision-making and understanding of illness. If the study results show that ACP is effective, this will promote its uptake among health care providers and patients both within Singapore and in other countries.

### Limitations

Attrition may be a problem. We will try to minimize this through appropriate patient incentives and by scheduling interviews at times and places convenient for patients. It is also not possible to execute a double-blinded trial of ACP given that ACP is provided by a multidisciplinary team. Unblinded trials may be prone to bias, such as in assessment of primary outcome. We will attempt to reduce subjectivity in assessment of the outcome by having more than one senior investigator in the study team to review the contents of the medical records and to assess compliance with patients’ wishes as stated in the ACP or the survey documents. Contamination of the control arm is also possible if patients in the control arm seek ACP outside of the trial, during the follow-up phase.

### Strengths

The multidisciplinary research team brings the experience required to successfully complete the proposed study. The intervention to be evaluated (ACP) is one of the most promising interventions to promote conversations regarding EOL care between providers, patients and family members. Further, the ACP delivered is a standardized program developed based on the “Respecting Choices Program.” The facilitators are well trained and the environment for program delivery is conducive because of support from hospital staff, physicians and the government.

Lastly, we will also examine the reasons for success or failure of the ACP program in meeting the aims through qualitative in-depth interviews with health care staff and family members/nominated surrogates of the deceased. This will enable us to identify the strengths and limitations of ACP. Thus, the study is expected to have large potential benefit to society and may ultimately lead to improved clinical approaches to the care of patients with advanced CHF at the EOL.

## Trial status

The trial has been approved and is currently recruiting.

## Abbreviations

ACP, advance care planning; CHF, congestive heart failure; CPR, cardiopulmonary resuscitation; EOL, end-of-life; HADS, Hospital Anxiety and Depression Scale; KCCQ, Kansas City Cardiomyopathy Questionnaire
